# Co-occurrence of ecologically equivalent cryptic species of spider wasps

**DOI:** 10.1098/rsos.160119

**Published:** 2016-08-24

**Authors:** Hiroaki Kurushima, Jin Yoshimura, Jeong-Kyu Kim, Jong-Kuk Kim, Yutaka Nishimoto, Katsuhiko Sayama, Manabu Kato, Kenta Watanabe, Eisuke Hasegawa, Derek A. Roff, Akira Shimizu

**Affiliations:** 1 Department of Biological Sciences, Graduate School of Science and Engineering, Tokyo Metropolitan University, Hachioji, Tokyo 192-0397, Japan; 2 Graduate School of Science and Technology, and Department of Mathematical and Systems Engineering, Shizuoka University, Hamamatsu 432-8561, Japan; 3 Department of Environmental and Forest Biology, State University of New York College of Environmental Science and Forestry, Syracuse, New York 13210, USA; 4 Marine Biosystems Research Center, Chiba University, Kamogawa, Chiba 299-5502, Japan; 5 Bureau of Ecological Research, National Institute of Ecology, Seocheon, Chungcheongnam, 33657, Republic of Korea; 6 Department of Forest Environment Protection, College of Forest and Environmental Sciences, Kangwon National University, Chuncheon, Kangwon 24341, Republic of Korea; 7 10-1 Oharano-Taniue, Takarazuka, Hyogo 669-1211, Japan; 8 Hokkaido Research Center, Forestry and Forest Products Research Institute, Sapporo, Hokkaido 062-8516, Japan; 9 2-2-B205 Hirafuku, Tsuyama, Okayama 708-0872, Japan; 10 Science and Technology Division, Okinawa College, National Institute of Technology, Nago, Okinawa 905-2192, Japan; 11 Department of Botany, University of Hawaii at Manoa, Honolulu, Hawaii 96822, USA; 12 Laboratory of Animal Ecology, Department of Ecology and Systematics, Graduate School of Agriculture, Hokkaido University, Sapporo 060-8589, Japan; 13 Department of Biology, University of California, Riverside, CA 92521, USA

**Keywords:** coexistence, competitive exclusion principle, cryptic species, reproductive isolation, sympatric species

## Abstract

Many cryptic species have been discovered in various taxonomic groups based on molecular phylogenetic analyses and mating experiments. Some sympatric cryptic species share equivalent resources, which contradicts the competitive exclusion principle. Two major theories have been proposed to explain the apparent lack of competitive exclusion, i.e. niche-based coexistence and neutral model, but a conclusive explanation is lacking. Here, we report the co-occurrence of cryptic spider wasp species appearing to be ecologically equivalent. Molecular phylogenetic analyses and mating experiments revealed that three phylogenetically closely related species are found sympatrically in Japan. These species share the same resources for larval food, and two of the species have the same niche for nesting sites, indicating a lack of competitive exclusion. This evidence may suggest that ecologically equivalent species can co-occur stably if their shared resources are sufficiently abundant that they cannot be over-exploited.

## 1. Introduction

Phylogenetically closely related species complexes with almost no morphological distinctions are designated as cryptic species. Based on minute differences in pheromones and/or sexual behaviour, cryptic species have been detected in various taxonomic groups, e.g. moths and frogs [1]. Recently, molecular phylogenetic analyses have revealed many cryptic species in several animal taxa, especially in insects [1]. Moreover, mating experiments have confirmed that some of these cryptic species are reproductively isolated [2]. Cryptic species have been reported to be distributed allopatrically, parapatrically [3] or even sympatrically [1]. These sympatric species occasionally exhibit minor differences in their ecological niches [4]. Surprisingly, a few cryptic species exhibit no apparent differences in their ecological niches but still are distributed sympatrically over much of their distributions [5,6]. These findings appear to contradict the competitive exclusion principle [7–10].

The competitive exclusion principle states that species limited by a common resource cannot coexist [7–10]. It means that the principle is only applicable for species that are limited by common resources. In other words, the principle does not apply to species that share a common resource too abundant to be a limiting factor. Coexistence of two or more species are possible either with niche differentiation among the species [6,11] or if they share the unlimited resources. By contrast, neutral models are built on the assumption that all species are identical in their fitness and in their effects on one another. Population dynamics is driven randomly in births, deaths and dispersal, and multiple species are distributed sympatrically [12,13]. Neutral dynamics, however, cannot act to maintain species in a system, but rather only slow the loss of species from local communities [14]. Ecologically equivalent species should randomly go extinct as their relative abundances vary stochastically until only one species remains [13]. This means that under the neutral models, species exhibit temporal co-occurrence, but do not maintain stable coexistence.

*Auplopus carbonarius* (Scopoli, 1763) is a solitary hunting wasp of the family Pompilidae. This species is widely distributed in the temperate zone of the Palearctic Region, from Europe to the Far East [15–17]. The female builds barrel-shaped mud cells and stores a small spider in each cell [17,18]. The larva feeds on the spider and grows in the cell.

**Figure 1.**
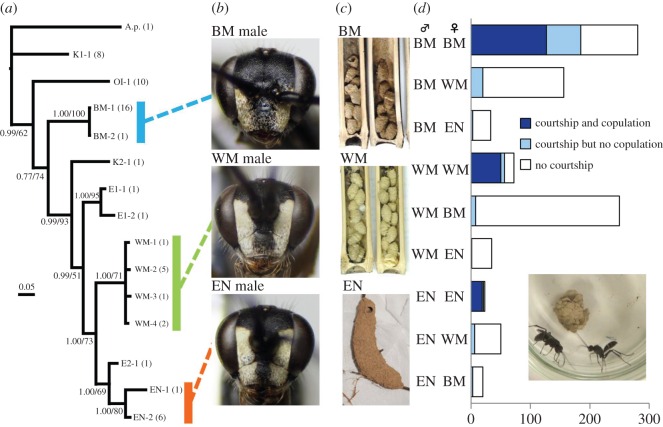
Three cryptic species of the *Auplopus carbonarius* species complex. (*a*) Phylogenetic tree based on BI analyses of COI and 28S datasets combined (terminal taxa, haplotypes; numbers in parentheses, sample sizes). (*b*) Male heads of the three cryptic species, frontal view. (*c*) Nests of the three species. (*d*) Results of the mating experiments. The three wasp types are BM: black-mandibular type; EN: exposed nesting type and WM: white-mandibular type. A.p.: *Auplopus pygialis* (Pérez, 1905) (outgroup); K1: Korean type 1; OI: Okinawa Island type; K2: Korean type 2; E1: European type 1; E2: European type 2. The two numbers on each branch represent the Bayesian posterior probabilities (greater than or equal to 0.5) and ML bootstrap supports (greater than or equal to 50). The copulation success of males with conspecific females is significantly more frequent than that with non-conspecific females (for statistics, see the electronic supplementary material, figure S5). The courtship of males toward conspecific females is significantly more frequent than that toward non-conspecific females (for statistics, see the electronic supplementary material, figure S5).

The Japanese *A. carbonarius* consists of three morphological/behavioural types, i.e. the white-mandibular type (WM type), black-mandibular type (BM type) [15,19] and exposed nesting type (EN type) [20] (figure 1*b* and electronic supplementary material, figure S1). In the male of the WM type, the mandible is ivory-white on its greater part and the frons has a pair of lateral longitudinal ivory-white maculae extending above the level of the antennal sockets. In the male of the BM type, the mandible is wholly black or dark brown and the frons has a pair of ivory-white maculae not extending above the level of the antennal sockets. The male of the EN type is indistinguishable from that of the WM type in head coloration. In the female, there are no morphological differences among the three types. The females of both WM and BM types construct nests in closed spaces such as the insides of plant stems, hollow spaces under the bark and gaps in stone walls. The EN-type female, however, builds nests on exposed substrates such as the undersides of leaves or tree roots hanging down under overhang earth cliffs, and the nest cells are covered with additional mud sheets [20] (figure 1*c*).

Here, we report a case of study of stable co-occurrence of ecologically equivalent cryptic species for which another plausible explanation seems to be applicable. We present data on the co-occurrence of three cryptic species of a spider wasp species complex (figure 1). First, based on molecular phylogenetic analyses and mating experiments, we confirmed that the three species are reproductively isolated. Then, we demonstrated that these species are widely distributed sympatrically throughout Japan based on collection records and trap nest surveys. Among these species, two use the same nesting site and all three share a common prey spider species as larval food. From this evidence, we propose a new hypothesis: ecologically equivalent species can stably co-occur if they share resources that are sufficiently abundant to not be over-exploited.

## 2. Material and methods

### 2.1. Collection and rearing of wasps

The WM- and BM-type wasps were collected by using bamboo tube nest traps [21–23]. The traps consisted of dried bamboo stems approximately 200 mm long and 10 mm in internal diameter, with one end closed by the node. Ten bamboo stems were tied together with two cable ties and were attached horizontally to tree trunks approximately 1.5 m above the ground. The traps were set up at 33 localities in Japan (electronic supplementary material, table S4). Each mud cell in the bamboo tubes was numbered and kept individually in a glass vial (20 ml) at the outdoor temperature in Hachioji-shi, Tokyo (mean temperature per month from October to December of 2010–2014: 6.9°C; from January to April of 2011–2015: 9.8°C). Adult wasps that emerged from mud cells were kept in the vials, being fed with solution of honey in water, and were used for mating experiments. Nests of the EN type were collected in 2012–2015 at two localities in Japan (electronic supplementary material, table S4). Each of them was numbered and kept individually in a small plastic container until adults emerged. Adults were moved to glass vials immediately after their emergence and were reared in the same way as described above. When both male and female wasps emerged from the same bamboo tube, we assigned the type of the male(s) to the female(s). This is justified because a bamboo tube is usually used by a single female [23,24] and consequently all wasps emerging from the same tube are considered to be siblings.

### 2.2. Examined materials for molecular analyses

For molecular analyses, we sampled the WM-, BM- and EN-type wasps that had already been used for mating experiments (electronic supplementary material, table S5). One of the mid legs was removed from an emerging wasp and preserved in 100% ethanol for DNA extraction, and the remainder of the wasp was kept as a voucher. We also analysed specimens of the *A. carbonarius* from Okinawa Island, Korea, France and England (electronic supplementary material, figure S1 and table S5). Male wasps from the Okinawa Island type (OI) are similar to those of the BM type in head coloration. In the Korean males, two morphological types are distinguishable by their head coloration, although both are similar to the WM type: the Korean type 1 (K1), has two small white spots on the frons between the antennal sockets, whereas in the Korean type 2 (K2) such spots are absent. In the males from France and England, other two morphological types are distinguishable based on head coloration: the European type 1 (E1) from France and England is similar to the WM type and the European type 2 (E2) from France is similar to the BM type. We added a female of *A. pygialis* (Pérez, 1905) collected from Saitama Prefecture of Japan to our materials as an outgroup.

### 2.3. Molecular analyses

We analysed two regions of the DNA: 595 bp of the mitochondrial cytochrome oxidase subunit I DNA (COI) and 497 bp of the nuclear 28S ribosomal DNA (28S). The COI, 28S and their combined datasets were analysed from the wasps shown in the electronic supplementary material, table S5.

The total DNA was extracted from one of the middle legs of each wasp that had been preserved in 100% ethanol at −20°C. The leg was incubated overnight at 55°C in 100 µl of Chelex 10% TE buffer containing 5 µl of proteinase K (20 mg ml^–1^). Subsequently, the samples were heated at 100°C for 10 min to deactivate the enzyme. The COI and 28S regions were amplified using the COI primers LCO-EG: 5′-TTTCAACAAATCACAAAGAYATYGG-3′ and HCO-EG: 5′-TAAACTTCAGGRTGACC RAAAAATCA-3′ [25,26] and the 28S primers D2B: 5′-GTCGGGTTGCTTGAGAGTGC-3′ and D3A-r: 5′-TCCGTGTTTCAAGACGGGTC-3′ [27]. Each PCR contained 5 µl of 2 × PCR buffer, 2 µl of dNTPs (final 0.4 mM), 0.3 µl of 10 pmol µl^−1^ forward and reverse primers (final 0.3 µM), 0.2 µl of 1.0 U µl^−1^ KOD FX Neo (TOYOBO Co., Ltd., Osaka, Japan) and 0.5 µl of DNA template. Thermal cycle parameters were as follows: 94°C for 2 min; 5 cycles of denaturation at 98°C for 10 s, 45°C for 30 s, 68°C for 45 s; then: for COI, 40 cycles at 98°C for 10 s, 48.5°C for 30 s, 68°C for 45 s; for 28S, 45 cycles at 98°C for 10 s, 51°C for 30 s, 68°C for 45 s, and the final extension at 68°C for 7 min. After confirming the PCR amplification on a 2.0% agarose gel, the amplified products were incubated at 37°C for 30 min and 80°C for 20 min with Illustra™ ExoStar (GE Healthcare, Buckinghamshire, UK) to remove any excess primers and nucleotides. The cycle sequencing reactions were run with ABI PRISM BigDye Terminator Cycle Sequencing Kit v. 3.1 (Applied Biosystems, Foster City, CA, USA). The sequencing reaction products were purified and concentrated by ethanol precipitation with sodium acetate. DNA sequencing was performed on an ABI PRISM 3130 Genetic Analyzer.

The obtained sequences have been deposited in the DDBJ database (electronic supplementary material, table S5). Sequences were edited by Chromas Pro 1.7 (Technelysium Pty. Ltd., South Brisbane, Queensland, Australia) and were aligned by MUSCLE [28] in MEGA 5.2 [29] with the Auto setting.

### 2.4. Phylogenetic inference

Maximum-likelihood (ML) and Bayesian inference (BI) analyses were run for the COI and 28S markers separately, as well as for the combined dataset. Nucleotide substitution models for the two molecular markers and the combined dataset were determined in Kakusan4 v. 4.0 [30]. The protein coding regions COI were partitioned by codon positions.

In the BI analyses, models were selected based on the Bayesian information criterion [31]; they were HKY + Gamma applied to the codon first and third positions of the COI dataset and the COI region of the combined dataset; F81 + Homogeneous applied to the codon second positions of the COI dataset and the COI region of the combined dataset; K80 + Gamma applied to the 28S region of the combined dataset and K80 + Homogeneous applied to the 28S dataset. In the ML analyses, the GTR + Gamma model was chosen for all datasets using the AICc [32]. We performed the BI analysis using MrBayes v. 3.1. 2 [33]. This analysis was conducted with two independent runs. Four chains were run for 1 000 000 generations each, and sampled every 100 generations. The first 1000 trees were discarded as burn-in. We used Tracer v. 1.6.0 [34] and checked if all parameter values of the runs became steady states, the posterior probability densities were similar between the runs and the effective sample size of parameter values exceeded 100. For the ML analysis, we applied the likelihood ratchets algorithm as a tree searching strategy using RAxML, v. 8.1.15 [35]. Bootstrap test was 1000 repeats for the ML.

To recognize species from our phylogenetic data objectively, we used a species delimitation method, the Bayesian Poisson tree process (bPTP) model [36] using the BI and ML trees of the COI and 28S datasets. The analysis was conducted using the online web service: http://species.h-its.org/ptp [36].

### 2.5. Mating experiments

A female and a male were paired in a petri dish (46 mm in diameter, 18 mm in height), and their behaviour was recorded for 10 min with digital video cameras (Canon, iVIS HFS21; SONY, Handycam HDR-XR500).

Nine hundred and twenty mating experiments were conducted at 9.00–18.00 of April–May of 2011–2015 and August of 2014 at Systematic Zoology laboratory at Tokyo Metropolitan University, using 606 wasps (electronic supplementary material, tables S1 and S4). These wasps were used for multiple mating experiments, except for mated females, because the female exhibits single mating as do females of most solitary wasps and bees [37]. Females were paired with heterotypic males prior to homotypic males. The average number of mating experiments per female was shown in the electronic supplementary material, table S6. The frequency of mating was also checked for both males and females. The results of mating experiments were tested statistically by Fisher's exact test with Bonferroni correction.

Ninety-nine females that were mounted by males and appeared to succeed in mating were dissected to confirm whether they had sperm in the spermatheca. This organ was removed from the females killed in the freezer and the presence or absent of sperm was checked under a microscope.

### 2.6. Comparison of ecological niche

We examined collecting sites and methods (bamboo trap nests or others, e.g. netting, yellow pan traps and Malaise traps), sex and morphological types of 4378 specimens deposited in the insect collection of Tokyo Metropolitan University. These data include the information on the wasps used for molecular analyses and mating experiments. Their type was identified by the method stated in ‘Collection and rearing of wasps'. We also examined prey spider remains in nests of all types (WM- and BM-type nests collected from trap nests at Minami-osawa, Hachioji-shi, Tokyo and EN-type nests collected from Shimo-oyamada-machi, Machida-shi, Tokyo). These two localities are only *ca* 4 km apart.

## 3. Results

### 3.1. Confirmation of separation among three cryptic species

Molecular phylogenetic analyses based on the COI and 28S markers indicate that the WM, BM and EN types are separated into monophyletic groups (figure 1*a*). These analyses also demonstrated five additional monophyletic groups: the OI type, Korean types 1 and 2 (K1 and K2), European type 1 (E1 from France and England) and European type 2 (E2 from France) (figure 1*a*). Furthermore, the bPTP analysis presented the possibility that these types are independent species, although support values for some species were not high (electronic supplementary material, figures S2 and S3).

Mating experiments were performed among the WM, BM and EN types (figure 1*d* and electronic supplementary material, figure S4 and table S1). Successful mating was observed only between homotypic pairs. A few male courtship trials were observed in heterotypic pairs, but none of them led to successful mating. For all three species, the copulation success and courtship between homotypic pairs were significantly more frequent than those between heterotypic pairs (electronic supplementary material, figure S5).

### 3.2. Comparison of distribution and ecological niche

**Figure 2.**
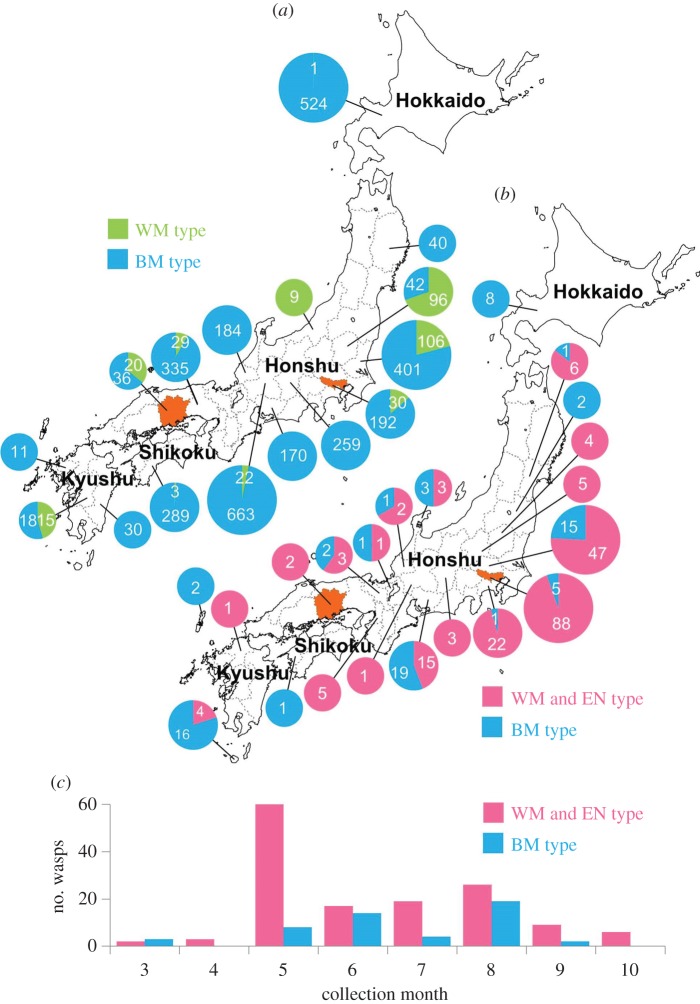
Distributions and periods of collection in the *Auplopus carbonarius* species complex. (*a*) Number of wasps collected with bamboo tube traps. (*b*) Number of wasps collected with methods other than bamboo tube traps, e.g. netting, yellow pan traps and Malaise traps. Pie charts are graded to three sizes according to the sample number. Orange-coloured areas indicate prefectures in which all three wasp types were collected. (*c*) Frequency distributions of collection dates on a monthly basis for the samples used in (*b*). Wasp types: BM, black-mandibular type; EN, exposed nesting type; WM, white-mandibular type. No significant differences are found in the collection months between the WM-EN combined and BM in (*c*) (Mann–Whitney *U*-test, *p* > 0.05).

The three types are distributed sympatrically throughout the Japanese mainland (figure 2*a,b*). Bamboo trap collections showed that both WM and BM types were collected from 9 out of 16 prefectures (figure 2*a* and electronic supplementary material, table S2). In other collections, WM (or EN) types and BM types were collected from 10 out of 21 prefectures (figure 2*b* and electronic supplementary material, table S3). We were not able to find any tendency in the distribution of the types. In Tokyo and Okayama Prefecture, all three types were collected in close proximity (less than 4 km). Thus, we conclude that these types are sympatrically distributed throughout the Japanese mainland.

As indicated by the trap nest records (figure 2*a* and electronic supplementary material, table S2), the same nest sites were shared by the WM and BM types in 13 localities. In addition, detailed records showed that these two types were found in the same 15 bamboo tube bundles. Among 254 tubes with two or more males hatching, two bamboo tubes produced both WM- and BM-type males. Moreover, a single tube was shared by both WM and BM types, which was confirmed by morphological and DNA analyses.

The active periods of adult males were also compared by the collection dates of samples by the methods other than bamboo tube traps (figure 2*c*). The records indicate that the active period of adult males was from March to October for the three types, although we could not separate the WM and EN types, because they cannot distinguish by morphological characters. The wasps collected with the trap nest method also exhibited no difference in the emergence periods among the three types (electronic supplementary material, figure S6).

**Figure 3.**
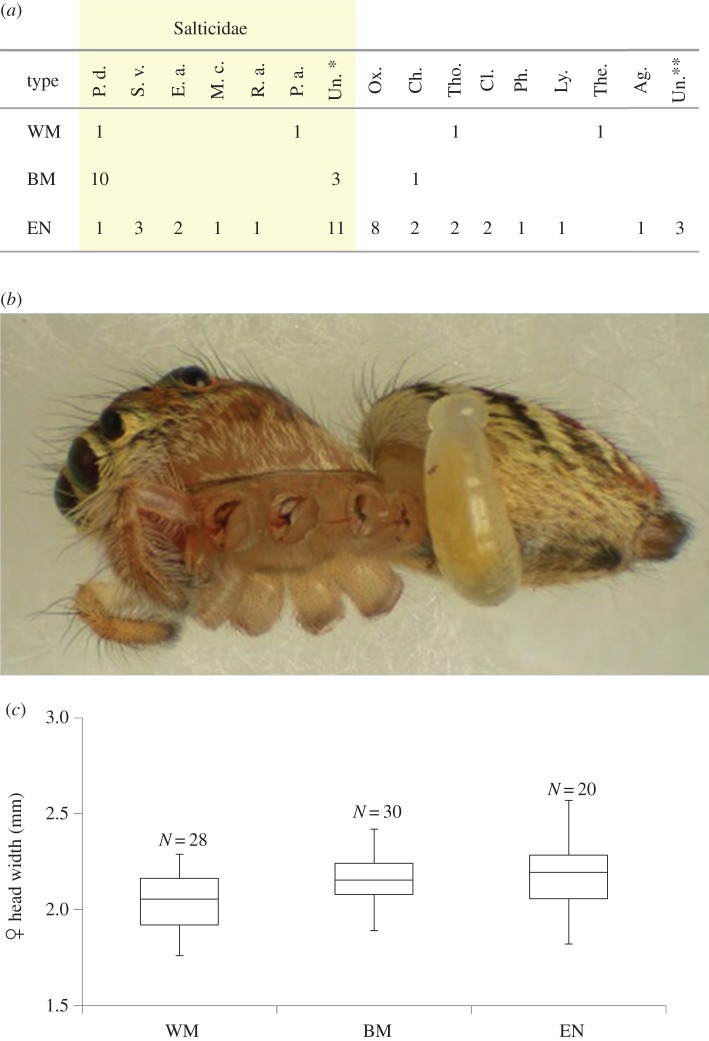
Prey spiders of the *Auplopus carbonarius* species complex. (*a*) Species and families of the prey spiders. (*b*) Photograph of the common prey *Plexippoides doenitzi* (Karsch, 1879) fed upon by a larva of *A. carbonarius* species complex. (*c*) Frequency distributions of head widths of the three types of females (median, upper/lower qualities, maximum and minimum). The WM female width was significantly smaller than those of the other two types (Mann–Whitney *U*-test with Bonferroni correction, *p* < 0.05), but range of the three types is largely overlapped. Ag., Agelenidae; Ch., Chiracanthiidae; Cl., Clubionidae; E. a., *Evarcha albaria* (Koch, 1878); Ly., Lycosidae; M. c., *Mendoza canestrinii* (Ninni, 1868); Ox., Oxyopidae; P. a., *Phintella abnormis* (Bösenberg & Strand, 1906); P. d., *Plexippoides doenitzi* (Karsch, 1879); Ph., Philodromidae; R. a., *Rhene atrata* (Karsch, 1881); S. v., *Siler vittatus* (Karsch, 1879); The., Theridiidae; Tho., Thomisidae; Un.*, unknown species of Salticidae; Un.**, unknown species.

Next, we examined the remains of the spider prey in the nest cells of the WM and BM types (in Minami-osawa, Hachioji-shi, Tokyo) and in the nest cells of the EN type (in Shimo-oyamada-machi, Machida-shi, Tokyo, *ca* 4 km from the former locality). The remains showed that the larvae of all the wasp types fed on small spiders of several families (figure 3*a*). The most frequent prey item was jumping spiders (Salticidae), among which *Plexippoides doenitzi* (Karsch, 1879) was preyed upon by all three wasp species (figure 3*a,b*). Finally, we examined the head width of the three types as an indicator of body size. The WM type was significantly smaller than the other two types, but their ranges largely overlapped (figure 3*c*).

## 4. Discussion

Our phylogenetic analyses differentiated the three phenotypes of *A. carbonarius* into three distinct monophyletic groups (figure 1*a*). These types were delimited as potential species by the bPTP analysis (electronic supplementary material, figures S2 and S3). In addition, the mating experiments revealed a strict behavioural isolation among these three types (figure 1*d* and electronic supplementary material, figure S5). Therefore, because of their genetic differentiation and reproductive isolation, we conclude that these organisms are separate species and form the *Auplopus carbonarius* species complex (we hereafter change ‘WM, BM and EN types’ to ‘species WM, BM and EN’). The molecular data and bPTP analyses also showed that wasps from Okinawa Island, Korea, France and England form five monophyletic groups that differ from the above three species (figure 1*a* and electronic supplementary material, figures S2 and S3). The two types from Korea (K1 and K2) and the two types from France (E1 and E2) were collected in the same localities, indicating the co-occurrence of each pair of types. Probably the *A. carbonarius* species complex comprises many cryptic species, which should be confirmed by further mating experiments.

The collection records from the bamboo tube traps and other methods suggest that at least the species WM and BM are widely distributed in the Japanese mainland (figure 2*a*). Because wasps of species EN are collected from Tokyo and Okayama Prefecture, their distributional area probably overlaps with those of species WM and BM.

In the three species, the emergence and active periods of adult males occurred from March to October with no significant differences among the species (figure 2*c*). The voltinism of these species is estimated to be two or more generations starting in April (electronic supplementary material, figure S6). Thus, we did not find differences in the emergence and active periods among the three species.

Here, we consider the niche overlap in nesting sites and prey food. The collection records obtained by the use of trap nests showed that species WM and BM share the same nesting sites, whereas species EN exhibits niche separation. Because these species are phylogenetically closely related (figure 1*a*), the separation of nesting niche might have led to the speciation of species EN from species WM. In addition, the two types from Korea share the same bamboo trap bundles and the two types from France were collected in the same locality with a Malaise trap.

Regarding the niche for prey food, the three wasp species hunt small spiders from several families (figure 3*a*). Among them, one of the most common targets is the jumping spiders (Salticidae), including *P. doenitzi*, which was preyed on by all three species. The correlation of body size among spider wasps (Pompilidae) and their prey spiders is well known [38]. We found that the body size of the wasp females is almost equal (figure 3*c*). Hence, the prey size of the three species is considered to be almost the same. These patterns suggest that the niche for prey is almost identical among the three species. It is necessary to validate this statistically. However, neither prey species composition nor prey abundance remains technically difficult to estimate. Further studies will be needed to confirm the identity of their prey utilization.

Any ecological differences, including ‘stabilizing niche differences’ [11], were not observed in our results. Three species have sympatric distributions and share a common niche for prey items, i.e. jumping spiders. Species WM and BM also share common nesting sites. We consider a possibility that species WM and BM speciated and avoid gene flow by reproductive differences (e.g. sex pheromones) and are ecologically equivalent to each other. Our ecological data showed only indirect suggestions, and the density variation, niche equivalency and ‘invasibility criterion’ [11,14,39] of each species were not verified. However, the neutral model [13] is unlikely to match this case because two species of wasps are widely and sympatrically distributed in Japan and either species does not converge to either 0 (extinction) or 1 (exclusion).

We here propose a new hypothesis. These wasps may coexist because of sharing of unlimited common resources. The common prey resources for the wasps of the three species are small, free-living spiders that far exceeded their predators' needs. The common nest resources for species WM and BM are small, closed spaces that are abundant compared with their overall requirements. In our trap nest bundles, a few tubes were almost always vacant, indicating that there are sufficient nesting sites for these wasps. Thus, the abundance of these resources exceeds the wasps' resource requirements. Certainly, there should be no competitive exclusion for these abundant resources. At present, we cannot prove this hypothesis because of lack of quantitative data on their food, nesting and other ecological niches. We will need to collect these kind of data for prolonged periods.

The abundance of resources is a key factor to avoid competitive exclusion. This raises an important question: why are these resources so abundant? The studied species have a low number of progeny because they have evolved to lay only a limited number of eggs in their lifetime and are K-strategists [40,41]. These small spider wasps hunt a relatively small spider on which they lay a single egg, e.g. the *Auplopus carbonarius* species complex. Because the small spiders hunted by these wasps are abundant compared with the needs of the wasp populations, the wasps do not deplete their resources. This lack of resource limitation results in the absence of competitive interactions among the wasp species that use the same resource.

We cannot verify the proposed hypothesis of unlimited resources from the current data, but these data help us to deduce this logical interpretation of coexistence of species. Owing to the difficulty of observations and experiments, the confirmation of the current hypothesis is likely to be impossible in this case, but may become possible using other examples. We should note that the proposed ‘coexistence’ should be termed ‘stable co-occurrence’ if the term ‘coexistence’ is defined solely by niche differentiation or the competitive exclusion principle [6,11]. Here, this stable co-occurrence is strictly different from the temporal co-occurrence of the neutral models [12,13].

## Supplementary Material

Supplementary Information

## Data accessibility

The data reported in this paper are tabulated in the electronic supplementary materials. DNA sequences were deposited in DDBJ (accession nos. LC090862 to LC090902, LC091212 to LC091256 and LC154073 to LC154113).

## References

[1] Bickford D, Lohman DJ, Sodhi NS, Ng PKL, Meier R, Winker K, Ingram KK, Das I 2007 Cryptic species as a window on diversity and conservation. Trends Ecol. Evol. 22, 148–155. (doi:10.1016/j.tree.2006.11.004) 17129636 10.1016/j.tree.2006.11.004

[2] Dincă V, Wiklund C, Lukhtanov VA, Kodandaramaiah U, Norén K, Dapporto L, Wahlberg N, Vila R, Friberg M 2013 Reproductive isolation and patterns of genetic differentiation in a cryptic butterfly species complex. J. Evol. Biol. 26, 2095–2106. (doi:10.1111/jeb.12211) 23909947 10.1111/jeb.12211 PMC4413813

[3] Vodă R, Dapporto L, Dincă V, Vila R 2015 Why do cryptic species tend not to co-occur? A case study on two cryptic pairs of butterflies. PLoS ONE 10, e0117802 (doi:10.1371/journal.pone.0117802) 25692577 10.1371/journal.pone.0117802 PMC4334660

[4] Gabaldón C, Serra M, Carmona MJ, Montero-Pau J 2015 Life-history traits, abiotic environment and coexistence: the case of two cryptic rotifer species. J. Exp. Mar. Biol. Ecol. 465, 142–152. (doi:10.1016/j.jembe.2015.01.016)

[5] McPeek MA, Gomulkiewicz R 2005 Assembling and depleting species richness in metacommunities: insights from ecology, population genetics and macroevolution. In Metacommunity ecology (eds Holyoak M, Leibold MA, Holt RD), pp. 355–373. Chicago, IL: University of Chicago Press.

[6] Leibold MA, McPeek MA 2006 Coexistence of the niche and neutral perspectives in community ecology. Ecology 87, 1399–1410. (doi:10.1890/0012-9658(2006)87[1399:COTNAN]2.0.CO;2) 16869414 10.1890/0012-9658(2006)87[1399:cotnan]2.0.co;2

[7] Hardin G 1960 The competitive exclusion principle. Science 131, 1292–1297. (doi:10.1126/science.131.3409.1292) 14399717 10.1126/science.131.3409.1292

[8] Hutchinson GE 1957 Concluding remarks. Cold Spring Harbor Symp. Quant. Biol. 22, 415–427. (doi:10.1101/SQB.1957.022.01.039)

[9] Hutchinson GE 1959 Homage to Santa Rosalia, or why are there so many kinds of animals? Am. Nat. 93, 145–159. (doi:10.1086/282070)

[10] MacArthur RH, Levins R 1967 The limiting similarity, convergence, and divergence of coexisting species. Am. Nat. 101, 377–385. (doi:10.1086/282505)

[11] Chesson P 2000 Mechanisms of maintenance of species diversity. Annu. Rev. Ecol. Syst. 31, 343–366. (doi:10.1146/annurev.ecolsys.31.1.343)

[12] Bell G 2000 The distribution of abundance in neutral communities. Am. Nat. 155, 606–617. (doi:10.1086/303345) 10777433 10.1086/303345

[13] Hubbell SP 2001 The unified neutral theory of biodiversity and biogeography. Princeton, NJ: Princeton University press. 10.1016/j.tree.2011.03.024 21561679

[14] Siepielski AM, McPeek MA 2010 On the evidence for species coexistence: a critique of the coexistence program. Ecology 91, 3153–3164. (doi:10.1890/10-0154.1) 21141177 10.1890/10-0154.1

[15] Tsuneki K 1990 Taxonomic notes on the Japanese species of *Auplopus* (Hymenoptera: Pompilidae). Spec. Publ. Jpn Hymennopterarists Assoc. 36, 66–80.

[16] Haupt H 1927 Monographie der Psammocharidae (Pompilidae), Mittel-, Nord- und Osteuropas. Dtsch Entomol. Z. 1926–1927, 1–367.

[17] Wahis R 2006 Mise à jour du Catalogue systématique des Hyménoptères Pompilides de la région ouest-européenne. Additions et Corrections. Notes Fauniques de Gembloux 59, 31–36.

[18] Day MC 1988 Spider wasps. Hymenoptera: Pompilidae. Handb. Identif. Br. Insects 6, 1–60.

[19] Haneda Y 1994 Pompiladae in Fukui Prefecture (3). Taxonomy of the genus *Auplopus*. Entomol. J. Fukui 15, 66–72.

[20] Iwata K 1975 From the field notes of a naturalist. Tokyo, Japan: Asahi Shimbun (in Japanese).

[21] Krombein KV 1967 Trap-nesting wasps and bees: life histories, nests and associates. Washington, DC: Smithsonian Institute Press.

[22] Matsumoto K, Makino S 2011 Monitoring of tube-nesting bees and wasps with bamboo tube nest traps of different types in two types of forests in temperate Japan. Entomol. Sci. 14, 154–161. (doi:10.1111/j.1479-8298.2010.00434.x)

[23] Shimizu A, Nishimoto Y, Makino S, Sayama K, Okabe K, Endo T 2012 Brood parasitism in two species of spider wasps (Hymenoptera: Pompilidae, *Dipogon*), with notes on a novel reproductive strategy. J. Insect Behav. 25, 375–391. (doi:10.1007/s10905-011-9298-0)

[24] Tsuneki K 1946 The Japanese hunting wasps: their ecology and psychology. Sapporo, Japan: Hoppo Shuppan-sha.

[25] Eguchi K, Bui VT, Oguri E, Maruyama M, Yamane S 2014 A new data of worker polymorphism in the ant genus *Dorylus* (Hymenoptera: Formicidae: Dorylinae). J. Asia-Pacific Entomol. 17, 31–36. (doi:10.1016/j.aspen.2013.09.004)

[26] Folmer O, Black M, Hoeh W, Lutz R, Vrijenhoek R 1994 DNA primers for amplification of mitochondrial cytochrome coxidase subunit I from diverse metazoan invertebrates. Mol. Mar. Biol. Biotechnol. 3, 294–299. 7881515

[27] Saux C, Fisher BL, Spicer GS 2004 Dracula ant phylogeny as inferred by nuclear 28S rDNA sequences and implications for ant systematics (Hymenoptera: Formicidae: Amblyoponinae). Mol. Phylogenet. Evol. 33, 457–468. (doi:10.1016/j.ympev.2004.06.017) 15336679 10.1016/j.ympev.2004.06.017

[28] Edgar RC 2004 MUSCLE: multiple sequence alignment with high accuracy and high throughput. Nucleic Acids Res. 32, 1792–1797. (doi:10.1093/nar/gkh340) 15034147 10.1093/nar/gkh340 PMC390337

[29] Tamura K, Peterson D, Peterson N, Stecher G, Nei M, Kumar S 2011 MEGA5: molecular evolutionary genetics analysis using maximum likelihood, evolutionary distance, and maximum parsimony methods. Mol. Biol. Evol. 28, 2731–2739. (doi:10.1093/molbev/msr121) 21546353 10.1093/molbev/msr121 PMC3203626

[30] Tanabe AS 2011 Kakusan4 and Aminosan: two programs for comparing nonpartitioned, proportional, and separate models for combined molecular phylogenetic analyses of multilocus sequence data. Mol. Ecol. Resour. 11, 914–921. (doi:10.1111/j.1755-0998.2011.03021.x) 21592310 10.1111/j.1755-0998.2011.03021.x

[31] Schwarz G 1978 Estimating the dimension of a model. Ann. Statist. 6, 461–464. (doi:10.1214/aos/1176344136)

[32] Sugiura N 1978 Further analysts of the data by Akaike' s information criterion and the finite corrections. Commun. Statist. Theor. Meth. 7, 13–26. (doi:10.1080/03610927808827599)

[33] Ronquist F, Huelsenbeck JP 2003 MrBayes 3: Bayesian phylogenetic inference under mixed models. Bioinformatics 19, 1572–1574. (doi:10.1093/bioinformatics/btg180) 12912839 10.1093/bioinformatics/btg180

[34] Rambaut A, Suchard MA, Xie D, Drummond AJ 2014 Tracer v1.6, software distributed by the author at http://beast.bio.ed.ac.uk/Tracer.

[35] Stamatakis A 2014 RAxML version 8: A tool for phylogenetic analysis and post-analysis of large phylogenies. Bioinformatics 30, 1312–1313. (doi:10.1093/bioinformatics/btu033) 24451623 10.1093/bioinformatics/btu033 PMC3998144

[36] Zhang J, Kapli P, Pavlidis P, Stamatakis A 2013 A general species delimitation method with applications to phylogenetic placements. Bioinformatics 29, 2869–2876. (doi:10.1093/bioinformatics/btt499) 23990417 10.1093/bioinformatics/btt499 PMC3810850

[37] Thornhill R, Alcock J 2001 The evolution of insect mating systems. Lincoln, UK: iUniverse Inc.

[38] Kurczewski FE, Kurczewski EJ 1968 Host records for some North American Pompilidae (Hymenoptera) with a discussion of factor in prey selection. J. Kansas Entomol. Soc. 41, 1–33.

[39] Cothran RD, Noyes P, Relyea RA 2015 An empirical test of stable species coexistence in an amphipod species complex. Oecologia 178, 819–831. (doi:10.1007/s00442-015-3262-1) 25680335 10.1007/s00442-015-3262-1

[40] Iwata K 1955 The comparative anatomy of the ovary in Hymenoptera. Part I. Aculeata. Mushi 29, 17–34.

[41] Ito Y 1981 Comparative ecology. Cambridge, UK: Cambridge University Press.

