# Supplementary material for: Co-occurrence of ecologically equivalent cryptic species of spider wasps
Source: R Soc Open Sci. 2016 Aug 24;3(8):160119. doi: 10.1098/rsos.160119 (PMC5108940; doi:10.1098/rsos.160119)
Supplement: Supplementary Information [file rsos160119supp1.pdf]

Supplementary Information

Co-occurrence of ecologically equivalent cryptic species of spider wasps

Hiroaki Kurushima, Jin Yoshimura, Jeong-Kyu Kim, Jong-Kuk Kim, Yutaka Nishimoto, Katsuhiko Sayama, Manabu Kato, Kenta Watanabe, Eisuke Hasegawa, Derek A. Roff, and Akira Shimizu

- Supplementary Results
- Supplementary Discussion
- Fig. S1. Head of *Auplopus carbonarius* species complex, anterior view (**A**-**K**) and whole body of its female (**L**).
- Fig. S2. Phylogenetic tree based on Bayesian inference analyses of COI dataset of Auplopus carbonarius species complex and results of bPTP analysis.
- Fig. S3. Phylogenetic tree based on Bayesian inference analyses of 28S dataset of Auplopus carbonarius species complex and results of bPTP analysis.
- Fig. S4. Mating behavior of Auplopus carbonarius species complex (black mandibular type).
- Fig. S5. Statistical tests for mating experiments.
- Fig. S6. Per-month frequency distributions of wasps of Auplopus carbonarius species complex emerging from bamboo tube traps collected in winter.
- Fig. S7. Female's mate rejection to different type males.
- Table S1. Results of mating experiments.
- Table S2. Number of wasps collected by using bamboo tube traps.
- Table S3. Number of wasps collected by using methods other than bamboo tube traps.
- Table S4. Number of wasps in mating experiments for each locality.
- Table S5. Sample and haplotype numbers of molecular phylogenetic analyses.
- Table S6. Average number of mating experiments per female.
- Table S7. Pairwise-distances among COI haplotypes.
- Table S8. Pairwise-distances among 28S dataset of wasps.
- Movie S1. Mating behavior of black mandibular type of Auplopus carbonarius species complex, which comprises courtship, copulation, freezing and female's mate rejection.
- Movie S2. Female's mate rejection of white mandibular type of Auplopus carbonarius species complex.Fig. S2. Title of the second supplementary figure.

Supplementary Results

Molecular phylogenetic analyses

Haplotypes of each dataset and pairwise-distances among them are shown in table S7 and S8. In the BI and ML trees of the COI dataset (figure S2), the EN and WM types are not separated into monophyletic groups. In the COI dataset, maximum intratypic pairwise distance of the EN type was greater than the minimum pairwise distance between the WM and EN types (table S6). In contrast, the results of phylogenetic analyses based on the 28S dataset and mating experiments showed that they have been segregated at the species level. This contradiction suggests the possibility of mitochondrial gene introgression or incorrect phylogenetic relationships. Another study of Pompilidae also shows that the COI barcording region may produce misleading phylogenetic relationships [42].

The topology of the BI and ML trees of the 28S dataset is similar to that of the BI and ML trees of the combined dataset and suggests that the WM, BM and EN types (and also the OI, K1, K2, E1 and E2 types) are separated into each monophyletic group (figure S3).

We should also note that there are few haplotypes in all three species (Table S5), even though a fast region in COI is included in the current sequencing. Among them, the BM type is almost uniform: one sample (one haplotype) is different at one base pair from the other common haplotype, even though samples are collected widely from Hokkaido to Kyushu in Japan. Because samples are limited in corrected localities, this result may be due to the deviation of samples. We need further studies to find the reason for the scarcity of haplotypes in this species complex.

Mating experiments

(1) Normal mating behavior of each type

Between a female and a male of the same type, the following behavioral sequence was observed (figure S4 and Supplementary Movie S1): the male moved toward the female, raising the metasoma and vibrating the wings, and then mounted on her dorsum ("courtship behavior"). In almost all courtships of the WM type wasps (44/50), the male rushed to the female without wing vibration. In many courtships of the BM type wasps (93/127), however, the male directly leaped on the female, vibrating the wings. In all EN type courtships, the male rushed toward the female, and many of them vibrated the wings (13/19). Next, the male lowered its metasoma on the female, vibrating both wings and antennae, and attempted to insert the genitalia into the female vagina. The male repeated this action many times until successful copulation. When the male genitalia were inserted, the female stopped moving for a while ("freezing"). Most males, after copulation, exhibited further courtship behavior toward the females. The latter, however, drove off the former ("mate rejection") and a second copulation never occurred.

(2) Examination of sperms

In the females that had exhibited freezing (WM type: n = 24; BM type: n = 64; EN type: n = 1), sperm was found within the spermathecae. However, females not exhibiting freezing never had sperm (WM type: n = 3; BM type: n = 7). Based on this, we regarded other females exhibiting freezing as successful in mating.

(3) The frequency of male and female mating

Females of this species complex exhibit single mating in their lifetime as many other solitary wasps and bees [43]. All females having copulated exhibited mate rejection and never

copulated again even though the same type of males courted (WM type: n = 4; BM type: n = 20). In contrast, males often copulated multiple times (up to three times; WM type: n = 8; BM type: n = 24; EN type: n = 4).

(4) Characteristic mating behavior of each type

Some differences in mating behavior were found among the types.

(4-1) *Mounting time*

The WM and EN type males mounted on the same type females and kept the position more than one minute until they were driven off (mounting time: WM type, 141.7 sec., 62－238, SD = 46.3, n = 12; EN type, 131.9 sec., 72－258, SD = 64.1, n = 8). In contrast, the BM type males continued mounting for only several seconds (4.5 sec., 3–7, SD = 1.1, n = 17). The mounting time of the BM type was much shorter than that of the WM and EN types.

(4-2) *Female's mate rejection behavior*

The female's mate rejection behavior also varied among the types. Both the WM and EN type females rapidly turned around several times *in situ* when the same type males exhibited courtship behavior (Supplementary Movie S2). In contrast, the BM type females never turned around but moved away from the same type males or drove them off with the legs.

The "turning-around" behavior of virgin females was also found in the following heterotypic pairs: the WM type female and EN type male; and the EN type female and WM type male. This behavior was, however, never found when the BM type males courted the WM or EN type females.

(5) Female's mate rejection between different types

Males occasionally attempted to copulate with other type females and all of them failed (courtship: WM type, n = 9; BM type, n = 23; EN type, n = 9; mounting: WM type, n = 5; BM type, n = 10; EN type, n = 2). When the males mounted the females and attempted to insert their genitalia, the females appeared to close their metasomal apices (figure S7). In contrast, all type females opened it to copulate with the same type males.

Supplementary Discussion

Proximate cause of reproductive isolation

Because the rate of the male courtship between the same type wasps was significantly higher than that between the different type wasps, the cause of the reproductive isolation of the WM, BM and EN types is interpreted proximately as the male's selection of the same type female. It is unclear what factors caused the male's selection. It is likely the male was not able to distinguish female types by morphological or behavioral cues. Because all type females have almost the same morphology, including color, and females didn't appear to exhibit any special behavior before male's courtship. Males are likely to use invisible cues for the perception of the same type females. It is known that many cryptic animal species use nonvisual mating signals [1] and that many hymenopterous species use sex pheromones [43].

It is also probable that the female discriminates the same type male from different type males. Although some males courted other type females, they never copulated. Even when males mounted on the other type females and attempted to insert their genitalia, they never succeeded. When the female perceives the same type male, it may also be that the male sends out some signals to the female. Probably the signals are nonvisual because the WM and EN type males are very similar morphologically. There is a possibility that the male uses chemical signals. Male pompilids have the "Day's organ", which is situated on both the intersegmental membranes and the metasomal terga and is suspected to be secretory organs of some sex pheromones [18, 44]. Some males were observed to raise the metasoma (figure S4A), extend its segments and expose the intersegmental membranes when they courted. They may emit some pheromones from the organs and send them out by wing vibration. Alternatively, they might make courtship sounds with the wings as certain pteromalid and braconid males [45, 46]. It is, however, noteworthy that most males of the WM type courted without wing vibration. Because copulation never occurred when females were courted by heterotypic males, a female's mate selection is assumed to be more accurate than that of the male.

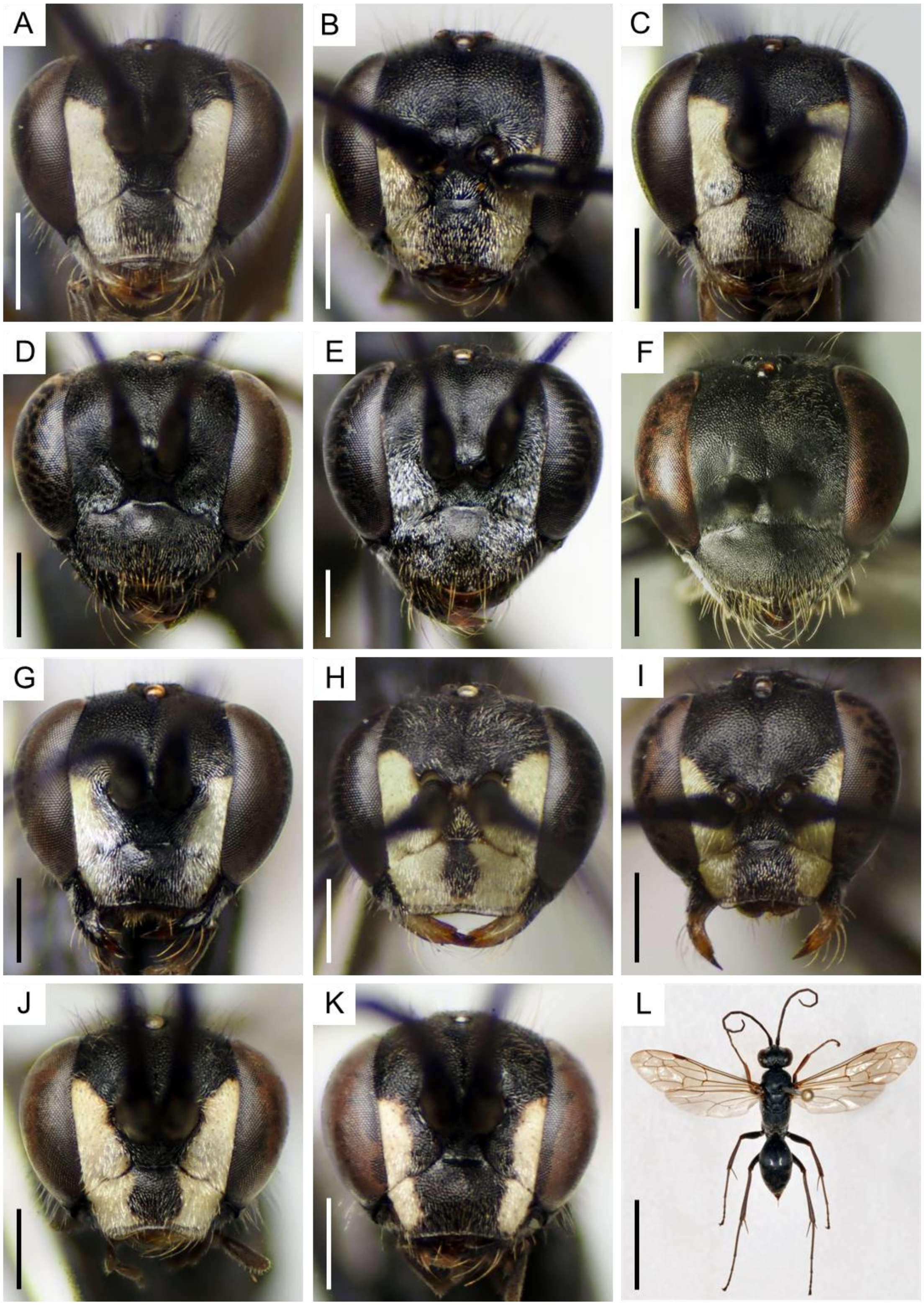

Supplementary Fig. S1: Head of *Auplopus carbonarius* species complex, anterior view (A-K) and whole body of its female (L). (**A**) White mandibular type male; (**B**) black mandibular type male; (**C**) exposed nesting type male; (**D**) White mandibular type female; (**E**) black mandibular type female; (**F**) exposed nesting type female; (**G**) Okinawa Island type male; (**H**) Korean type 1 male; (**I**) Korean type 2 male; (**J**) European type 1 male; (**K**) European type 2 male. Scales: 0.5 mm for (**A-K**); 5 mm for (**L**).

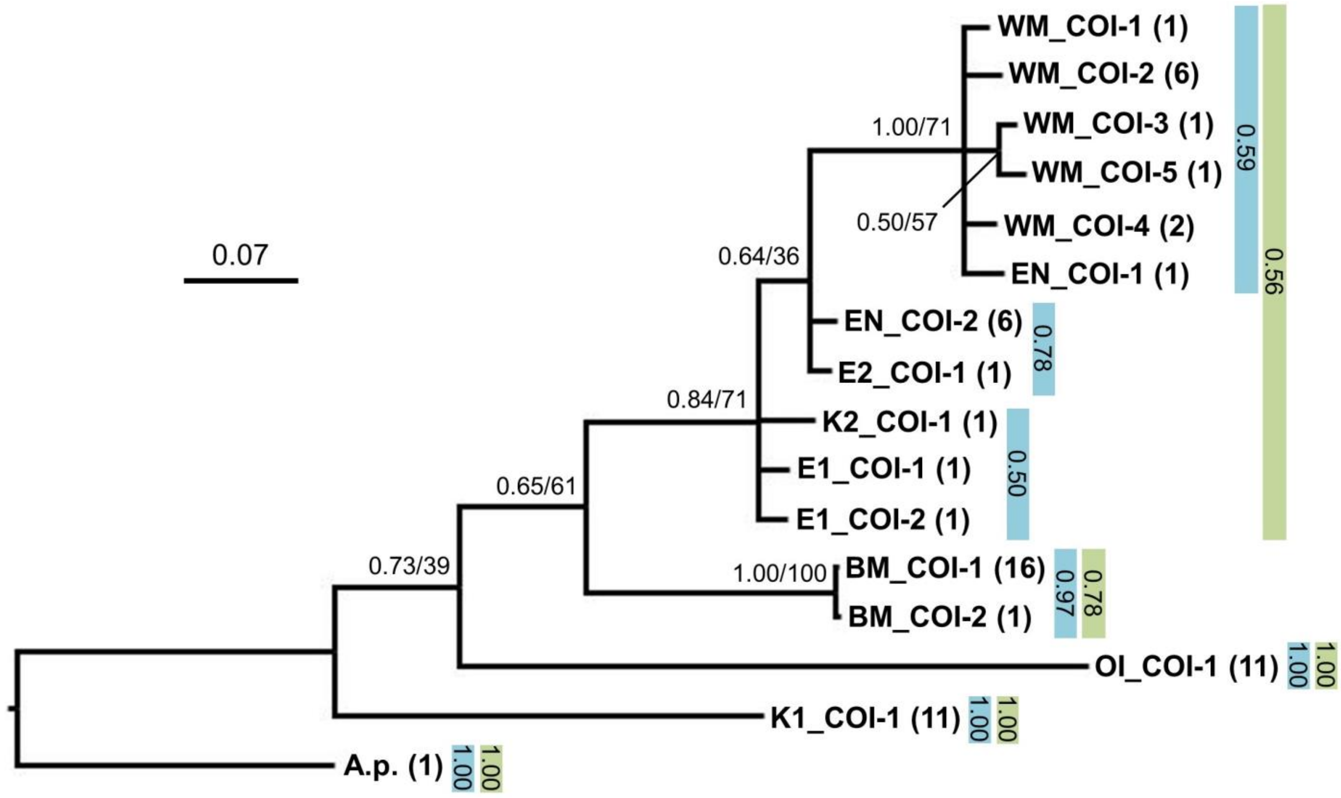

Supplementary Fig. S2: Phylogenetic tree based on Bayesian inference analyses of COI dataset of *Auplopus carbonarius* species complex and results of bPTP analysis. Two numbers on each branch represent Bayesian posterior probabilities (≥ 0.5) and Maximum likelihood bootstrap supports (≥ 50). Numbers in parentheses indicate sample sizes. Colored bars indicate delimited species by bPTP analysis based on both Bayesian inference and maximum likelihood trees (blue bars, Bayesian inference tree; green bars, maximum likelihood tree). Numbers in bars designate support values. A.p.: *Auplopus pygialis* (outgroup); BM_COI-1 and BM_COI -2: COI haplotypes of black mandibular type; E1_COI -1 and E1_COI -2: COI haplotypes of European type 1; E2_COI -1: COI haplotype of European type 2; EN_COI -1 and EN_COI -2: COI haplotypes of exposed nesting type; K1_COI -1: COI haplotype of Korean type 1; K2_COI -1: COI haplotype of Korean type 2; OI_COI -1: COI haplotype of Okinawa Island type; WM_COI - 1, WM_COI -2, WM_COI -3, WM_COI -4 and WM_COI -5: COI haplotypes of white mandibular type.

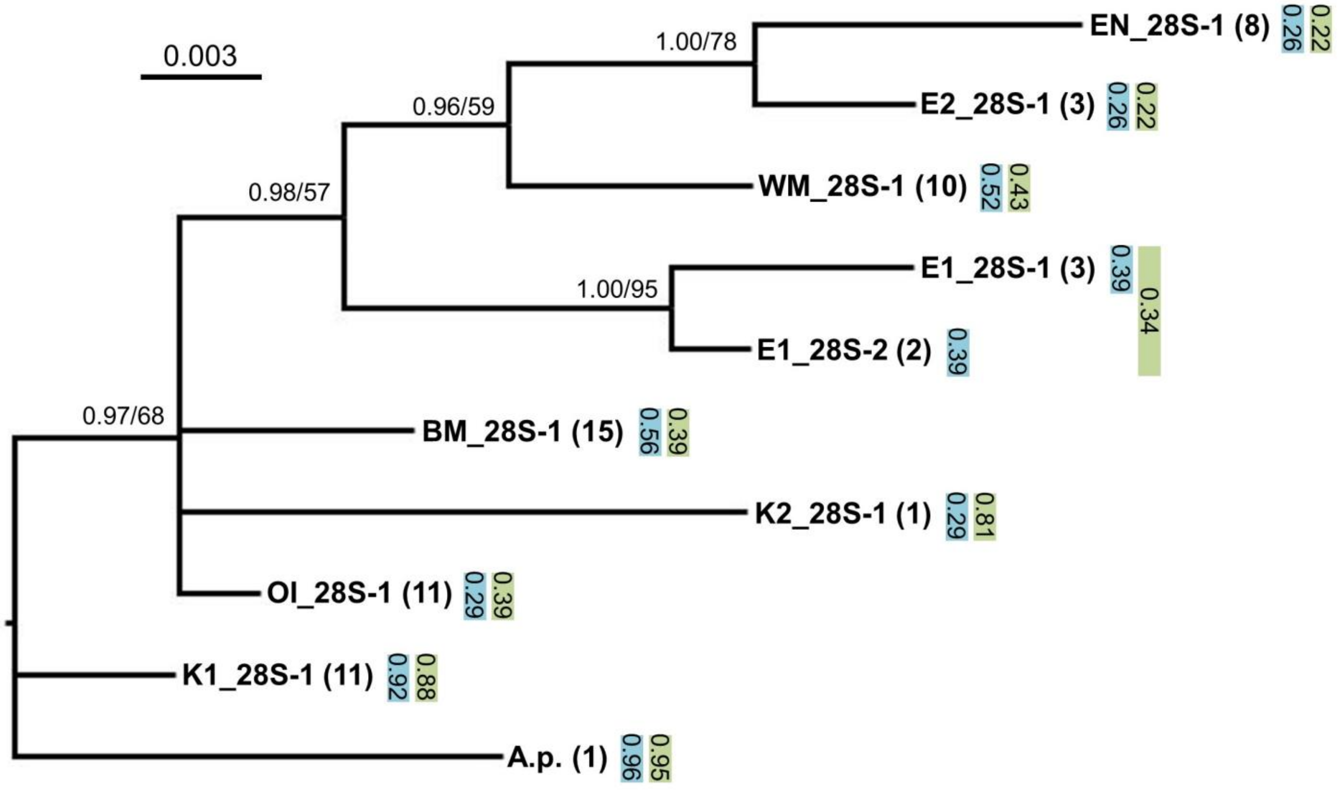

Supplementary Fig. S3: Phylogenetic tree based on Bayesian inference analyses of 28S dataset of *Auplopus carbonarius* species complex and results of bPTP analysis. Two numbers on each branch represent Bayesian posterior probabilities (≥ 0.5) and Maximum likelihood bootstrap supports (≥ 50). Numbers in parentheses indicate sample sizes. Colored bars indicate delimited species by bPTP analyses based on both Bayesian inference and maximum likelihood trees (blue bars, Bayesian inference tree; green bars, maximum likelihood tree). Numbers in bars designate support values. A.p.: *Auplopus pygialis* (outgroup); BM_28S-1: 28S haplotype of black mandibular type; E1_28S -1 and E1_28S -2: 28S haplotypes of European type 1; E2_28S -1: 28S haplotype of European type 2; EN_28S -1: 28S haplotype of exposed nesting type; K1_28S -1: 28S haplotype of Korean type 1; K2_28S -1: 28S haplotype of Korean type 2; OI_28S -1: 28S haplotype of Okinawa Island type; WM_28S -1: 28S haplotypes of white mandibular type.

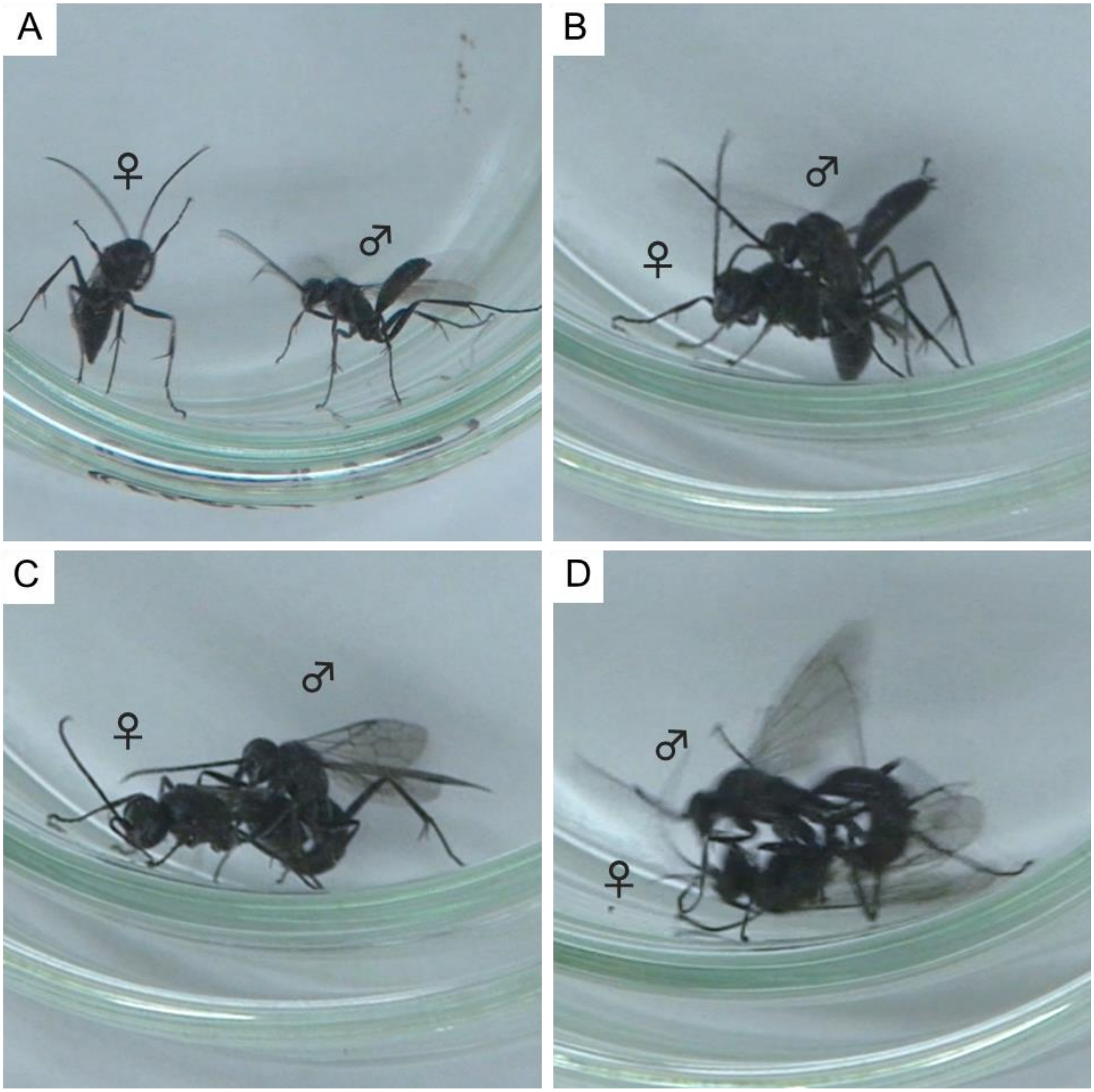

Supplementary Fig. S4: Mating behavior of *Auplopus carbonarius* species complex (black mandibular type). (**A**) Courtship behavior-1, male moving toward female, raising metasoma and vibrating wings; (**B**) courtship behavior-2, male mounting on female dorsum and attempting to insert genitalia into female vagina; (**C**) male inserting genitalia.; (**D**) mate rejection, female driving off male exhibiting further courtship behavior after copulation.

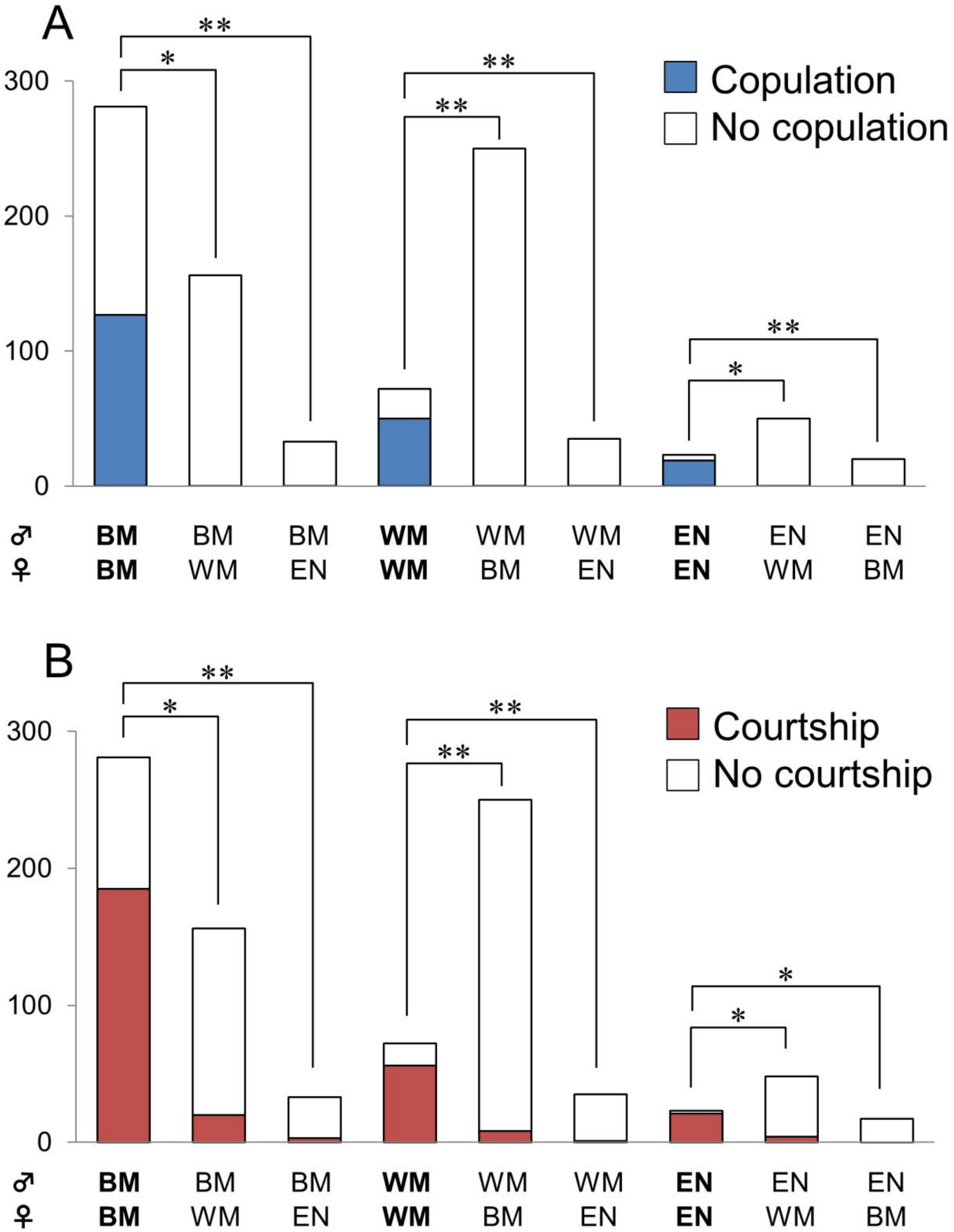

Supplementary Fig. S5: Statistical tests for mating experiments. (**A**) The copulation success of males with conspecific females is significantly more frequent than that with non-conspecific females (*, $P < 0.0000001$; **, $P < 0.000000000001$ in Fisher's exact test with Bonferroni correction). (**B**) The courtship of males toward conspecific females is significantly more frequent than that toward non-conspecific females (*, $P < 0.00000001$; **, $P < 0.0000000000001$ in Fisher's exact test with Bonferroni correction).

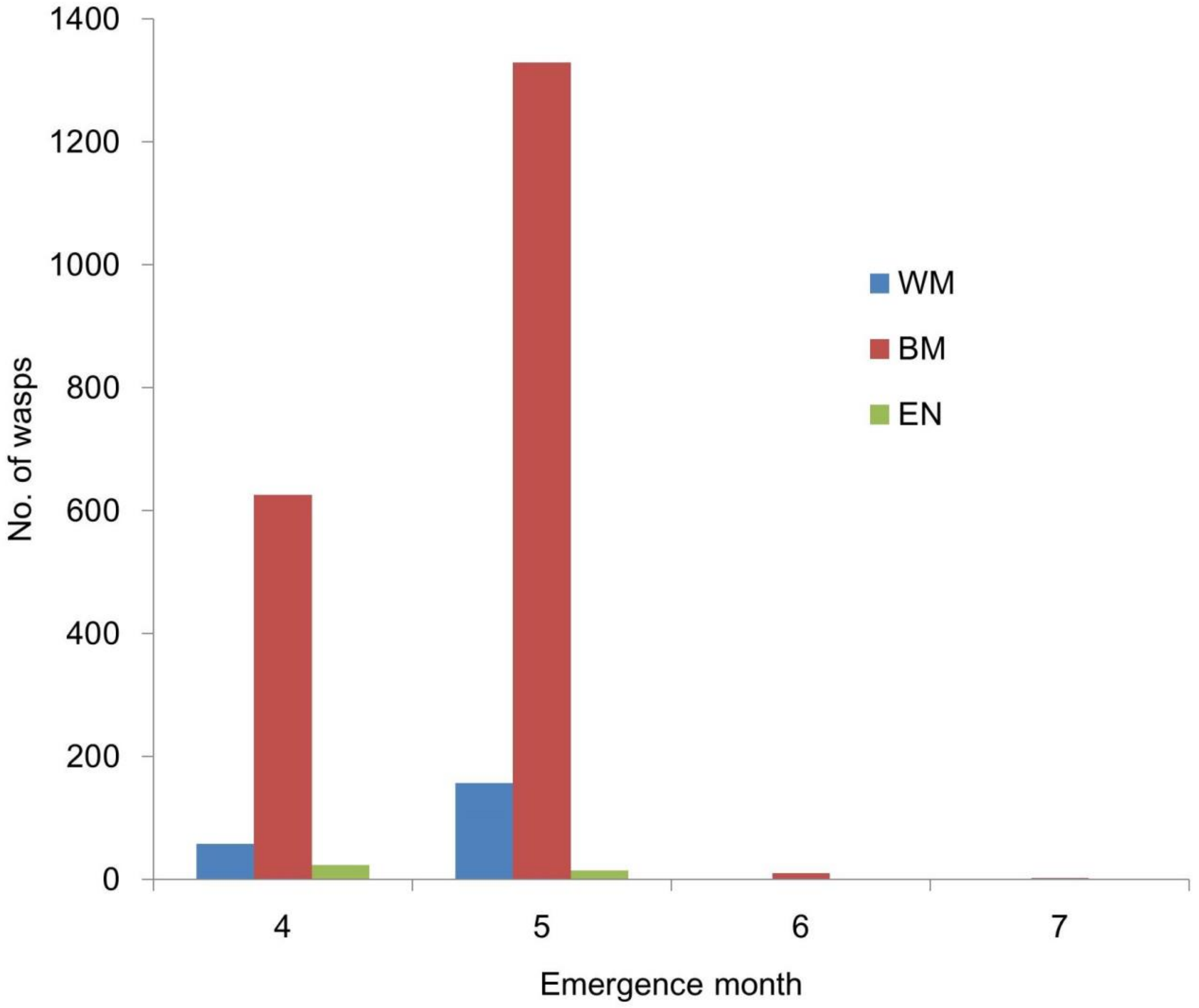

Supplementary Fig. S6: Per-month frequency distributions of wasps of *Auplopus carbonarius* species complex emerging from bamboo tube traps collected in winter. All wasps were considered the first generation. WM: White mandibular type male; BM: black mandibular type male; EN: exposed nesting type male. The main emergence period of all three type wasps was April–May.

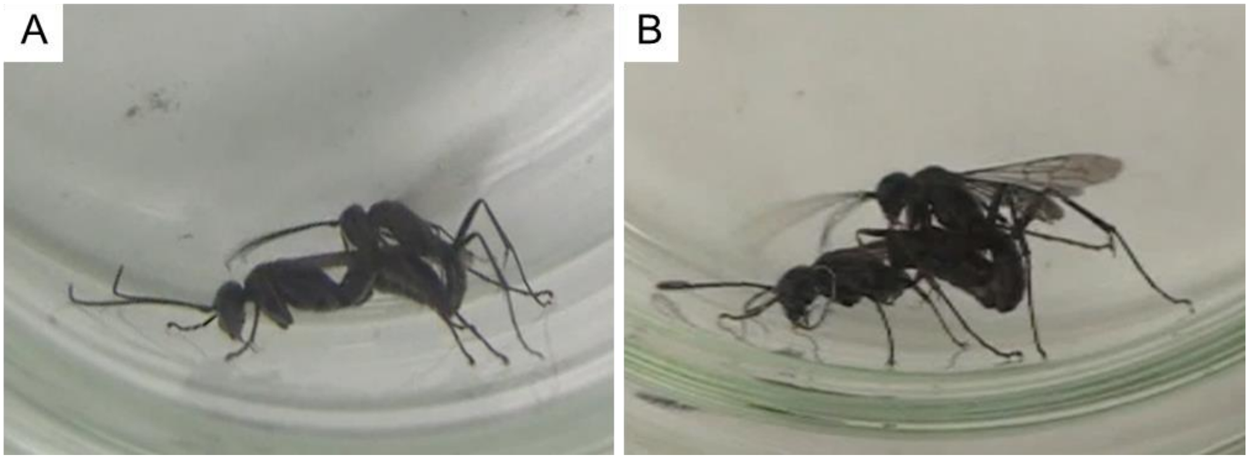

Supplementary Fig. S7: Female's mate rejection to different type males. (**A**) A black mandibular type female mounted by a white mandibular type male, the latter attempting to insert his genitalia and the former appearing to close her metasomal apex; (**B**) white mandibular type female opening their metasomal apex to copulate with the same type male.

Supplementary Table S1: Results of mating experiments. WM, white mandibular type; BM, black mandibular type; EN, exposed nesting type.

Type		Courtship			
Male	Female	Copulation	No copulation	No courtship	Totals
WM	WM	50	6	16	72
WM	BM	0	8	242	250
WM	EN	0	1	34	35
BM	BM	127	58	96	281
BM	WM	0	20	136	156
BM	EN	0	3	30	33
EN	EN	19	2	2	23
EN	WM	0	6	44	50
EN	BM	0	3	17	20
				Totals:	920

Supplementary Table S2: Number of wasps collected by using bamboo tube traps. WM, white mandibular type; BM, black mandibular type; *, Unknown because of the head missing.

Locality	WM		BM		Unknown	
	Males	Females	Males	Females	Males*	Females
Hokkaido	1		358	166		50
Iwate			17	23		7
Niigata	8	1				
Tochigi	53	43	20	22		5
Ibaraki	48	58	187	214		96
Tokyo	13	17	80	112		12
Fukui			85	99		27
Nagano			111	148	1	62
Aichi			113	57		17
Gifu	13	9	465	198	4	54
Hyogo	23	6	182	153	2	55
Okayama	12	8	19	17		
Kochi	2	1	170	119		1
Saga			7	4		
Miyazaki			11	19		5
Kumamoto	8	7	14	4	2	18
Totals:	181	150	1839	1355	9	409

Supplementary Table S3: Number of wasps collected by using methods other than bamboo tube traps. WM, white mandibular type; BM, black mandibular type; EN, exposed nesting type. EN type wasps were identified by their nests.

Locality	WM or EN	EN		BM	Unknown
	Males	Males	Females	Males	Females
Hokkaido	8				2
Miyagi	6			1	0
Fukushima				2	13
Tochigi	4				0
Gunma	5				2
Saitama	46			15	45
Tokyo	34	23	31	5	27
Kanagawa	22			1	3
Nagano	3				12
Aichi	15			19	35
Gifu	1				3
Ishikawa	3			3	
Fukui	2			1	
Shiga	1			1	3
Kyoto	3			2	5
Osaka	3	1	1		11
Okayama		1	1		
Ehime				1	1
Fukuoka	1				2
Nagasaki (Tsushima Island)				2	2
Kagoshima (Yakushima Island)	4			16	
Totals:	161	25	33	69	166

Supplementary Table S4: Number of wasps in mating experiments for each locality. WM, white mandibular type; BM, black mandibular type; EN, exposed nesting type.

Locality		WM		BM		EN	
		Males	Females	Males	Females	Males	Females
Hokkaido	Hitsujigaoka, Toyohira-ku, Sapporo-shi			77	59		
Iwate Pref.	Nabeyashiki, Shimokuriyagawa, Morioa-shi			11	18		
Niigata Pref.	Kariwa-mura, Kariwa-gun	8	1				
Tochigi Pref.	Kuzuri, Nakagawa-machi, Nasu-gun	43	32	7	18		
	Miwa, Nakagawa-machi, Nasu-gun	6	2	2	4		
Tokyo	Minami-osawa, Hachioji-shi	9	14	31	47		
	Shimo-oyamada-machi, Machida-shi					17	22
Hyogo Pref.	Kirihatanagaoyama, Takarazuka-shi			5	2		
Okayama Pref.	Tsukadani, Kagamino-cho, Tomata-gun	3	3	5	7	1	1
	Doi, Kagamino-cho, Tomata-gun	6	5	13	1		
	Tsukadani or Doi, Kagamino-cho, Tomata-gun	3					
Kochi Pref.	Nonogawa, Shimanto-shi			7	3		
	Nishitosamochii, Shimanto-shi			5	3		
	Nishitosafujinokawa, Shimanto-shi			5	4		
	Nishitosatachibana, Shimanto-shi			9	3		
	Kobushinokawa, Kurishio-cho, Hata-gun				2		
	Jujirou, Shimanto-shi			2			
	Nishitosanakaba, Shimanto-shi			1	1		
	Oyu, Shimanto-shi			3			
	Nishitosakuchihoriuchi, Shimanto-shi	2	1	3	2		
	Kobi, Shimanto-shi			2	1		
	Unoe, Shimanto-shi			3	1		
	Idanishinada, Kuroshio-cho, Hata-gun			2	5		
	Asou, Shimanto-shi			1			
	Iyono, Kozukushi-cho, Sukumo-shi			2	2		
	Shimonokae, Tosashimizu-shi			1	2		
	Souro, Tosashimizu-shi			2	1		
	Ueno, Tosashimizu-shi			2	1		
	Onotsumi, Tosashimizu-shi			1	3		
	Matsuo, Tosashimizu-shi			9	6		
	Ashizurimisaki, Tosashimizu-shi				1		
Saga Pref.	Sashi, Karatsu-shi			5	1		
	Nabatake, Karatsu-shi			1	3		
Totals:		80	58	217	210	18	23

Supplementary Table S5: Sample and haplotype numbers of molecular phylogenetic analyses. WM, white mandibular type; BM, black mandibular type; EN, exposed nesting type; OI, Okinawa Island type; K1, Korean type 1; K2 Korean type 2; E1, European type 1; E2, European type 2; A.p., *Auplopus pygialis* (outgroup). Numbers of haplotypes in parentheses.

Type	DNA region			DDBJ Accession No.	
	COI	28S	COI+28S	COI	28S
WM	11 (5)	10 (1)	9 (5)	LC090880, LC090886-LC090891, LC090899, LC090900, LC154093, LC154094	LC091212-LC091219, LC154073, LC154074
BM	17 (2)	15 (1)	13 (2)	LC090864-LC090879, LC090902	LC091227-LC091241
EN	7 (2)	8 (1)	7 (2)	LC090893, LC090901, LC154095-LC154099	LC091246-LC091248, LC154075-LC154079
OI	11 (1)	11 (1)	10 (1)	LC090894-LC090897, LC154107-LC154113	LC091249-LC091254, LC154088-LC154092
K1	11 (1)	11 (1)	8 (1)	LC090881-LC090884, LC154100-LC154106	LC091242-LC091244, LC154080-LC154087
K2	1 (1)	1 (1)	1 (1)	LC090885	LC091245
E1	2 (2)	5 (2)	2 (2)	LC090892, LC090898	LC091224-LC091226, LC091255-LC091256
E2	1 (1)	3 (1)	1 (1)	LC090863	LC091221-LC091223
A. p.	1 (1)	1 (1)	1 (1)	LC090862	LC091220
Totals:	62	65	52		

Supplementary Table S6: Average number of mating experiments per female. WM, white mandibular type; BM, black mandibular type; EN, exposed nesting type. Range of numbers of mating experiments is indicated in parentheses.

Female	Male		
	WM	BM	EN
WM	1.24 (0–5)	2.69 (0–8)	0.86 (0–7)
BM	1.20 (0–7)	1.35 (0–7)	0.10 (0–1)
EN	1.52 (0–9)	1.43 (0–6)	1.00 (0–2)

Supplementary Table S7: Pairwise-distances among COI haplotypes. WM, white mandibular type; BM, black mandibular type; EN, exposed nesting type; OI, Okinawa Island type; K1, Korean type 1; K2 Korean type 2; E1, European type 1; E2, European type 2; A.p., *Auplopus pygialis* (out group); WM-1, WM-2, WM-3, WM-4 and WM-5: COI haplotypes of WM type; BM-1, BM-2: COI haplotypes of BM type; EN-1, EN-2: COI haplotypes of EN type; OI-1: COI haplotype of OI type; K1-1: COI haplotype of K1 type; K2-1: COI haplotype of K2 type; E1-1, E1-2: COI haplotypes of E1 type; E2-1: COI haplotype of E2 type.

Type	COI haplotype	WM					BM		EN		OI	K1	K2	E1		E2
		WM-1	WM-2	WM-3	WM-4	WM-5	BM-1	BM-2	EN-1	EN-2	OI-1	K1-1	K2-1	E1-1	E1-2	E2-1
WM	WM-2	0.003														
	WM-3	0.002	0.005													
	WM-4	0.002	0.002	0.003												
	WM-5	0.003	0.007	0.002	0.005											
BM	BM-1	0.029	0.029	0.03	0.027	0.029										
	BM-2	0.03	0.03	0.032	0.029	0.03	0.002									
EN	EN-1	0.005	0.005	0.007	0.003	0.008	0.03	0.032								
	EN-2	0.01	0.01	0.012	0.008	0.014	0.029	0.03	0.012							
OI	OI-1	0.042	0.042	0.044	0.041	0.042	0.044	0.046	0.042	0.042						
K1	K1-1	0.029	0.029	0.03	0.027	0.029	0.035	0.037	0.03	0.029	0.044					
K2	K2-1	0.012	0.012	0.014	0.01	0.015	0.029	0.03	0.014	0.008	0.042	0.03				
E1	E1-1	0.012	0.012	0.014	0.01	0.015	0.029	0.03	0.014	0.005	0.042	0.03	0.007			
	E1-2	0.012	0.012	0.014	0.01	0.015	0.024	0.025	0.014	0.005	0.041	0.03	0.008	0.005		
E2	E2-1	0.008	0.008	0.01	0.007	0.012	0.027	0.029	0.01	0.002	0.041	0.027	0.007	0.003	0.003	
A. p.		0.035	0.032	0.037	0.034	0.039	0.047	0.049	0.035	0.035	0.047	0.041	0.032	0.034	0.035	0.034

Supplementary Table S8: Pairwise-distances among 28S dataset of wasps. WM, white mandibular type; BM, black mandibular type; EN, exposed nesting type; OI, Okinawa Island type; K1, Korean type 1; K2 Korean type 2; E1, European type 1; E2, European type 2; A.p., *Auplopus pygialis* as an out group; WM-1: 28S haplotype of WM type; BM-1: 28S haplotype of BM type; EN-1: 28S haplotype of EN type; OI-1: 28S haplotype of OI type; K1-1: 28S haplotype of K1 type; K2-1: 28S haplotype of K2 type; E1-1, E1-2: 28S haplotypes of E1 type; E2-1: 28S haplotype of E2 type.

Type	28S	WM	BM	EN	OI	K1	K2	E1		E2
	Haplotype	WM-1	BM-1	EN-1	OI-1	K1-1	K2-1	E1-1	E1-2	E2-1
BM	BM-1	0.01								
EN	EN-1	0.012	0.014							
OI	OI-1	0.008	0.002	0.012						
K1	K1-1	0.012	0.006	0.016	0.004					
K2	K2-1	0.02	0.014	0.02	0.012	0.016				
E1	E1-1	0.016	0.014	0.02	0.012	0.016	0.024			
	E1-2	0.012	0.01	0.016	0.008	0.012	0.02	0.004		
E2	E2-1	0.006	0.01	0.008	0.008	0.012	0.016	0.016	0.012	
A. p.		0.02	0.014	0.024	0.012	0.012	0.024	0.024	0.02	0.02
